# Supplementary material for: Hidden Markov models reveal behavioral state dynamics in depth-related locomotion in mice
Source: PLoS One. 2025 Aug 26;20(8):e0329367. doi: 10.1371/journal.pone.0329367 (PMC12380309; doi:10.1371/journal.pone.0329367)
Supplement: S1 Text — (DOCX) [file pone.0329367.s001.docx]

# Hidden Markov Models Reveal Complex Depth Processing in Mouse Visual Behavior

# ~Supplemental information~

### Impact of Data Thinning on Trajectory Characteristics

One key methodological consideration was determining the appropriate temporal resolution for our analysis. While our video recordings captured movement at 30 frames per second, analyzing such high-frequency data presented both computational challenges and the risk of capturing non-meaningful micro-movements. Through systematic analysis (Supplemental Figure 1), we found that sampling every third frame (10 fps) preserved essential behavioral dynamics while enabling analysis of longer behavioral sequences. This temporal resolution captures meaningful state transitions while filtering out high-frequency movement artifacts.

### Preliminary Mixture Model Analysis

Before implementing a full Hidden Markov Model, we conducted preliminary mixture model analyses to determine the appropriate complexity needed to represent the behavioral data. Our analyses demonstrated that both angular changes and step lengths in the mouse trajectories required multiple components to be adequately described. Angular data needed at least two components to capture both minor directional adjustments and broader directional shifts, while step length data required three components to represent low, intermediate, and high movement intensities (Supplemental Figure 2). While these mixture models did not define the exact number or transitions of hidden behavioral states, they clearly indicated the need for a multi-component approach and guided our selection of appropriate probability distributions for the emissions.

### Number of states

HMMs identify latent behavioral states corresponding to distinct movement patterns, with temporal dependencies incorporated to analyze transitions between states. Unlike simple mixture models, which consider only observed data distributions, HMMs allow for a more comprehensive analysis by including time-dependent patterns. This makes them particularly suitable for behavioral studies.

Based on our mixture model analysis, we determined that three states were necessary to adequately describe the data. A two-state model oversimplified behavioral patterns, failing to capture intermediate movement intensities, while a four-state model improved statistical fit but introduced an additional state that lacked physiological interpretability and correspondence to observed behavior. The final three-state model balanced statistical adequacy with biological relevance and simplicity. The states were ordered by movement intensity, with "Resting" representing low movement, "Exploring" representing moderate movement, and "Navigating" representing high movement.

The choice of state-dependent distributions for the HMM was guided by the earlier mixture model analysis. Step lengths exhibited a multimodal distribution that required at least three components, with the Gamma distribution providing the best fit. Similarly, angular changes showed bimodal characteristics that were well modeled using two components of the wrapped Cauchy or von Mises distributions. These findings supported the use of Gamma distributions for step lengths ($l_{t}$) and wrapped Cauchy distributions for angular data ($\theta_{t}$​), which effectively captured sharp peaks and heavy tails. These state-dependent distributions allowed for clustering movement patterns into meaningful behavioral states.

### Transition Probability Matrix (TPM)

A key advantage of HMMs is their ability to incorporate environmental covariates to model the transition probabilities dynamically. Instead of assuming static probabilities, the transition probabilities can be expressed as functions of covariates, allowing the HMM to account for how external factors influence state transitions. This is achieved by parameterizing the TPM using a multinomial logistic regression model:

$$\gamma_{ijt}=\frac{exp(exp(\beta_{ij}^{T}x_{t}))}{\sum_{k=1}^{n} exp(\beta_{ik}^{T}x_{t})}$$

where $\beta_{ij}$ is the coefficient vector for the transition from state $i$ to state $j$, and $x_{t}$ is a matrix of environmental covariates at time $t$.

We implemented a structured transition matrix in the HMM, restricting transitions to adjacent states. This means that the mice can transition from "Resting" to "Exploring" and from "Exploring" to "Navigating," but not directly from "Resting" to "Navigating." This structure mirrors observed mouse behavior, where mice typically increase their activity gradually rather than making abrupt transitions between inactivity and high activity.

$$\Gamma=\left[ \gamma_{11} \gamma_{12} \gamma_{13} \gamma_{21} \gamma_{22} \gamma_{23} \gamma_{31} \gamma_{32} \gamma_{33} \right]$$

This adjustment reduced the number of transitions to be estimated from nine (for a fully connected three-state model) to seven, significantly simplifying the model. Moreover, restricting transitions to adjacent states in the TPM reflects the natural progression of mouse activity levels, enhancing the model's biological interpretability.The structured transition matrix becomes even more advantageous in models with higher state counts, where the number of possible transitions grows exponentially. By limiting transitions to adjacent states, we ensure that the model remains computationally feasible and biologically interpretable, aligning well with the observed behavioral sequences of the mice.

### Covariate Encoding

From earlier analyses (Figure 1), it became evident that mouse behavior is heavily influenced by spatial characteristics of the experimental apparatus. In the square setup, movement patterns were strongly dictated by corner preferences, while in the circular visual cliff setup, mice predominantly followed the circular edge (Figure 1C). This behavior aligns with known tendencies of mice to avoid open areas and seek sheltered paths. To isolate the effect of the visual cliff on behavioral state transitions, it was necessary to account for these spatial influences by including additional covariates: distance to the edge, distance to the center, and distance to the cliff (Figure 2D).

To better represent the influence of these spatial features on behavior, raw distances were transformed into a measure of influence using a sigmoid (inverse logit) function. The transformation was defined as:

$$\eta_{f} = \frac{1}{1+exp(\beta_{f}(x-x_{f}))}$$

where $x$ represents the distance to the feature, $x_{f}$ specifies the distance at which the influence of a feature begins to significantly affect behavior, while $\beta_{f}$ determines how rapidly this influence increases as the mouse approaches the feature. This transformation maps distances to a $[0,1]$ interval, where values closer to 1 indicate stronger influence as the mouse nears the feature of interest, and values closer to 0 reflect weaker influence as the distance increases. The sigmoid function is particularly well-suited for behavioral modeling because it naturally captures the non-linear saturation of influence observed in mouse behavior. At small distances, the influence rapidly increases, but beyond a certain range, the effect diminishes, reflecting the biological intuition that proximity has a finite impact on behavior.

The parameters $x_{f}$ and $\beta_{f}$ determine the shape of the transformation. The threshold $x_{f}$ specifies the distance at which influence begins to change significantly, while the slope $\beta_{f}$, constrained to negative values, ensures that influence increases as distance decreases. Larger magnitudes of $\beta_{f}$ correspond to more abrupt changes in influence, whereas smaller magnitudes represent a more gradual transition. For example, in the context of our experimental setup (30 cm radius), a slope of $\beta=-1$ produces a relatively gradual increase in influence, while steeper slopes correspond to sharper transitions.

While the parameters for most spatial features were estimated directly from the data, the cliff parameters were fixed based on empirical observations and the experimental design. Specifically, the threshold for the cliff was set at $x_{cliff}=6cm$ with $\beta_{cliff}=-1$. The values were chosen based on observations that the cliff influence gradually increased as the mouse approached the cliff but showed little variation in behavior immediately adjacent to it. Conversely, once the mouse was a certain distance away from the cliff, the influence of the cliff appeared negligible. This behavior informed the choice of a threshold $x_{cliff}=6cm$ and slope $\beta_{cliff}=-1$ that reflect a gradual increase in influence near the cliff, with saturation occurring both close to and far from the cliff edge.

By encoding distances as influence values, this approach accounts for the graded effects of spatial features on mouse behavior in a biologically meaningful manner. These transformed covariates were then incorporated into the Hidden Markov Model (HMM) framework, enabling the dynamic modeling of how environmental factors drive transitions between behavioral states.

### Accounting for edge constraints

To further account for the physical constraint imposed by the enclosure walls, we introduced a bias to encourage movement along the edge when the mouse is near it. This modification builds on the previously defined edge influence ($\eta_{edge}$) and adjusts the angular direction of movement to reflect the physical barrier. Under normal conditions, the angle of movement at time $t$ ($\theta_{t}$) is modeled as:

$$\theta_{t}\sim wCauchy(\theta_{t-1}, \rho_{t})$$

where $\theta_{t-1}$ is the movement angle at the previous time step, and $\rho_{t} \in[0,1]$ is the concentration parameter. A low $\rho$value indicates a near-uniform distribution, where the direction of movement at $t$ is almost independent of $\theta_{t-1}$ while a $\rho$ value close to 1 suggests that the animal will continue moving in a similar direction.

To account for the physical constraint of the enclosure walls, we adjusted the expected movement direction ($\lambda_{t}$) when the mouse is near the edge:

$$\lambda_{t}= (1-\eta_{edge})\theta_{t-1}+ \eta_{edge}(\theta_{edge}\pm\frac{\pi}{2})$$

Here $\theta_{edge}\pm\frac{\pi}{2}$ is the angle tangent to the edge aligning the movement direction along the wall. The sign ($+$ or $-$) depends on whether the movement is clockwise or counterclockwise relative to the wall. The influence parameter $\eta_{edge}$, increases as the mouse approaches the edge, ensuring that near the wall, the mouse's movement direction is dominated by the enclosure boundary. The updated angle is then modeled as:

$$\theta_{t}\sim wCauchy(\lambda_{t}, \rho_{t})$$

Near the edges, where $\eta_{edge}\to1$, the movement direction is dominated by the tangent to the edge, reflecting the physical constraint imposed by the wall. This adjustment aligns the statistical model with the physical realities of the experimental setup, allowing it to better capture the observed movement patterns near the edge.

The concentration parameter ($\rho_{t}$​) plays a critical role in modeling the angular movement of the mouse, as it determines the degree to which the direction of movement ($\theta_{t}$) is influenced by the previous direction ($\theta_{t-1}$). A low value of $\rho$​ results in a near-uniform distribution of directions, indicating that the direction at time $t$ is almost independent of the direction at time $t-1$. Conversely, a high value of $\rho$​ (close to 1) implies strong directional persistence, with the mouse continuing in the same direction.

To better account for the influence of environmental features, the concentration parameter was dynamically adjusted at each time step ($t$) based on the previously defined influences ($\eta_{cliff, t}$, $\eta_{edge,t}$, and $\eta_{center, t}$​). Specifically, the concentration parameter was modeled as a weighted average:

$$\rho_{t}= \frac{\rho_{z}+\eta_{cliff,t}\rho_{cliff,z}+\eta_{edge,t}\rho_{edge,z}+\eta_{center,t}\rho_{center,z}}{1+\eta_{cliff,t}+\eta_{edge,t}+\eta_{enter,t}}$$

Where: $\rho_{z}$ is the base concentration parameter for state $z$, $\rho_{cliff,z}$, $\rho_{edge,z}$, and $\rho_{center,z}$ are state specific concentration parameters for the cliff, edge, and center influences, respectively, $\eta_{cliff, z}$, $\eta_{edge,z}$, and $\eta_{center, z}$, are the corresponding influence values at time $t$. This formulation ensures that the concentration parameter dynamically reflects the combined effects of environmental influences on directional movement. For example, when the mouse is near the edge ($\eta_{edge}\to1$), $\rho_{t}$ increases to reflect the physical constraint imposed by the edge, encouraging directional persistence along the wall. Similarly, near the cliff ($\eta_{cliff}\to1$), $\rho_{t}$ adjusts to reflect the increased influence of the cliff on movement behavior.

By allowing $\rho_{t}$​ to vary dynamically, the model captures the nuanced interactions between environmental features and behavioral states. This approach enhances the flexibility and biological relevance of the Hidden Markov Model, ensuring that it reflects the complex, context-dependent movement patterns observed in the visual cliff experiment.

### Hierarchical Structure of the Hidden Markov Model

The hierarchical structure of the model enables the simultaneous analysis of both group-level effects and individual variability. By treating individual parameters as deviations from group-level parameters, the model captures consistent behavioral patterns across mice while accounting for individual differences. This approach enhances the robustness and generalizability of the model.

Most parameters follow a similar formulation, where individual-level parameters ($p$) are modeled as deviations from group-level parameters ($p_{0}$​), and the variability is captured by a group-level variance parameter ($\sigma_{\rho_{0}}$​​):

$p\sim Normal(p_{0}, \sigma_{p_{0}}$)

This hierarchical framework is applied to several key components of the model. The first is the Transition Probability Matrix (TPM) coefficients ($\beta$), which describe how environmental covariates—such as distance to the cliff, edge, or center—influence transitions between behavioral states. These coefficients are modeled hierarchically, allowing for group-level effects to inform individual-level variability.

The second component includes state-dependent parameters for observations. Step length parameters are modeled using Gamma distributions, where the mean ($l_{m}$) and variance ($l_{v}$) describe the distribution of step lengths for each behavioral state. Additionally, angular concentration parameters ($\rho$) are used to account for directional persistence in movement. These parameters include state-specific base values ($\rho_{0}$) and feature-modulated values ($\rho_{cliff}, \rho_{edge}, \rho_{center}$) , reflecting the influence of environmental features on movement direction.

The final component addresses feature-specific effects, which capture the influence of proximity to environmental features such as the cliff, edge, or center on movement behavior. These effects are modeled through thresholds ($x_{f}$​) and slopes ($\beta_{f}$​) for sigmoid-transformed covariates, enabling the model to account for nonlinear spatial effects.

This hierarchical design offers several advantages. First, it allows for robust estimation of group-level parameters by pooling data across individuals, facilitating the identification of baseline effects specific to experimental groups. Second, the model accounts for individual-level variability by incorporating individual-specific deviations from group-level parameters, enabling the capture of behavioral differences within groups. Finally, the consistent parameterization of the hierarchical structure supports scalability, allowing the model to handle complex systems with multiple covariates and states efficiently while minimizing the risk of overfitting or excessive computational burden.

In summary, our HMM integrates observed movement data with environmental influences to infer underlying behavioral states. This model sets the stage for analyzing how mice interact with the visual cliff and how environmental features affect their behavioral transitions. Our implementation of a structured HMM with dynamic covariates and hierarchical modeling provides a nuanced understanding of mouse behavior in response to environmental features. This approach offers a framework that can be applied to other studies of animal movement and behavioral ecology.
