# Supplementary figures and images for: Hidden Markov models reveal behavioral state dynamics in depth-related locomotion in mice
Source: PLoS One. 2025 Aug 26;20(8):e0329367. doi: 10.1371/journal.pone.0329367 (PMC12380309; doi:10.1371/journal.pone.0329367)

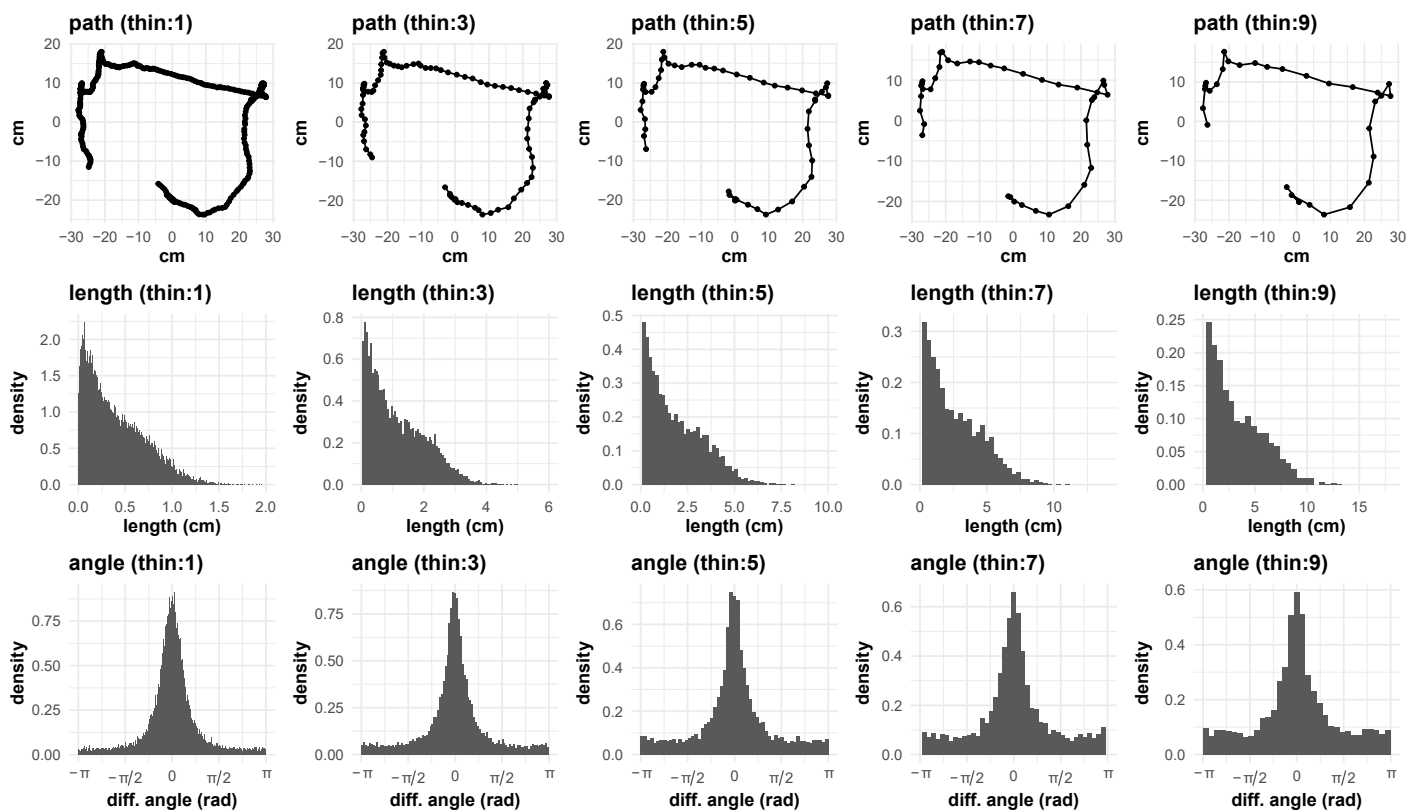

**Supplemental Figure 1**

Supplement: S1 Fig — (Top panels) Trajectory Plots: Example trajectories of a mouse subjected to different thinning levels. Thin 1 represents unthinned raw data at 30 fps, while Thin 3 and Thin 5 show trajectories sampled at effective frame rates of 10 fps and 6 fps, respectively. As thinning increases, finer details such as small, tight turns are omitted, resulting in smoother trajectories. (Middle panels) Step Length Distributions: Distributions of step lengths for each thinning level. The horizontal axis is adjusted to account for the increased time intervals due to thinning. While step lengths naturally increase with greater thinning, the overall shape of the distributions remains consistent, indicating that general movement dynamics are preserved. (Bottom panels) Angular Difference Distributions: Distributions of angular differences between consecutive steps for each thinning level. These distributions broaden with greater thinning, reflecting larger angular deviations due to the omission of finer trajectory details. This effect corresponds with the smoother paths observed at higher thinning levels. (PDF) [file pone.0329367.s002.pdf]

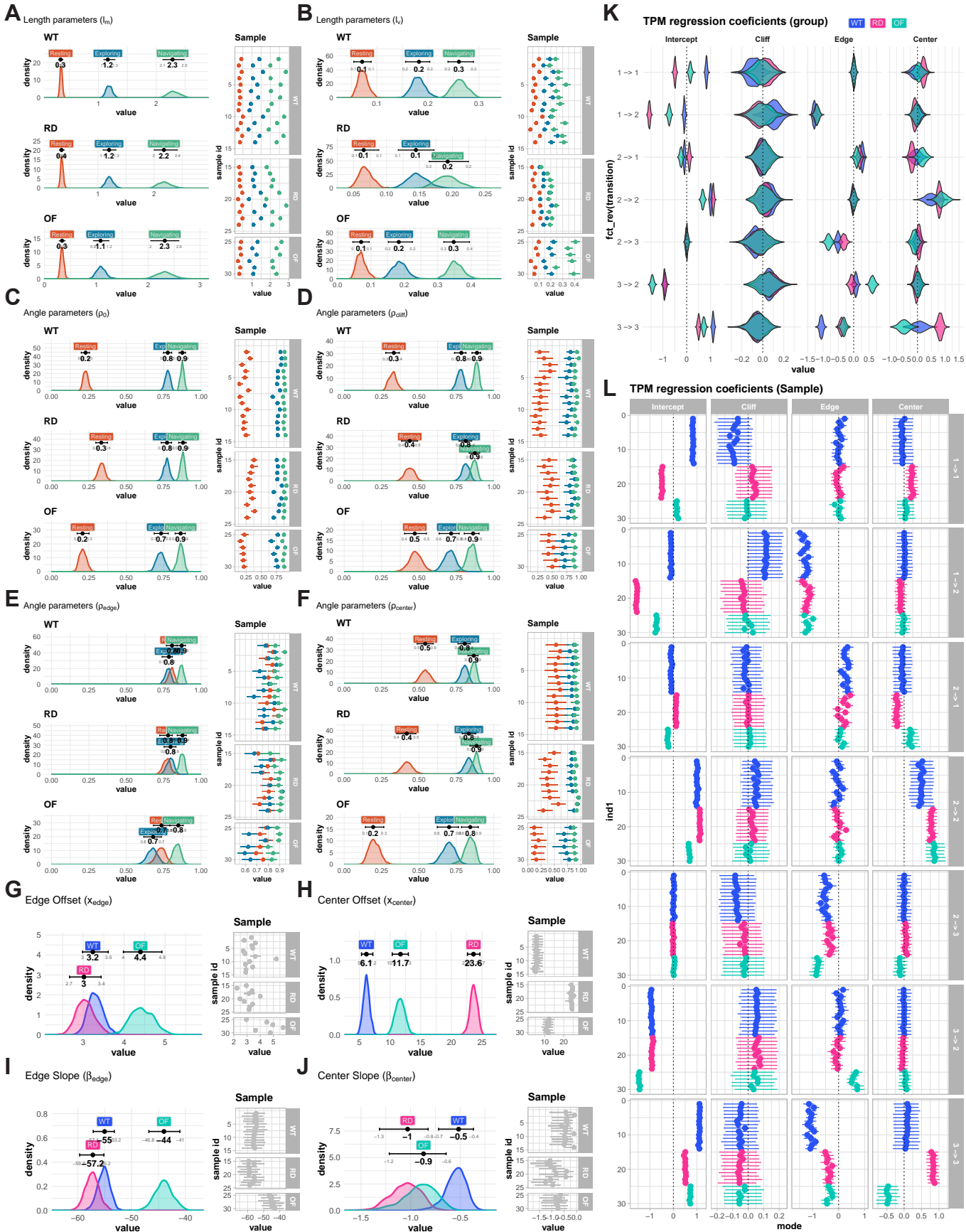

Supplemental Figure 4

Supplement: S4 Fig — (A, B) Step length parameters: Posterior distributions of the step length parameters for the three behavioral states (“Resting”, “Exploring” and “Navigating”) are shown for wild-type (WT), retinal degeneration (RD), and open field (OF) groups. WT mice exhibit distinct separation between states, while RD and OF groups show overlapping distributions, particularly for states 1 and 2, reflecting less structured movement patterns. (C ~ F) Angular concentration parameters: These parameters describe the directional persistence for each state under basal conditions and near environmental features (cliff, edge, center). WT mice display sharper directional persistence compared to RD and OF groups, particularly near the cliff, highlighting their reliance on visual depth cues. (G ~ J) Feature-specific influence parameters: Posterior distributions of the slope and offset parameters for center and edge influences are shown. WT mice demonstrate smaller center offsets compared to RD and OF groups, reflecting a stronger and more localized center influence. Edge parameters remain consistent across groups, underscoring the uniform effect of physical barriers. (K, L) Transition probability matrix (TPM) regression coefficients: Group-level and individual-specific posterior distributions of TPM regression coefficients are shown, highlighting the influence of spatial covariates (cliff, edge, center) on transitions between behavioral states. WT mice exhibit stronger spatial modulation in transitions, particularly between states 1 (“Resting”) and 2 (“Exploring”), while RD and OF mice show flatter distributions, indicating weaker transitions driven by spatial cues. This figure summarizes the posterior distributions of key HMM parameters, providing insights into how spatial features and behavioral states are modulated across WT, RD, and OF groups. The differences in posterior estimates underscore the distinct behavioral strategies employed by each group in response to the visual cliff apparat [file pone.0329367.s005.pdf]

A

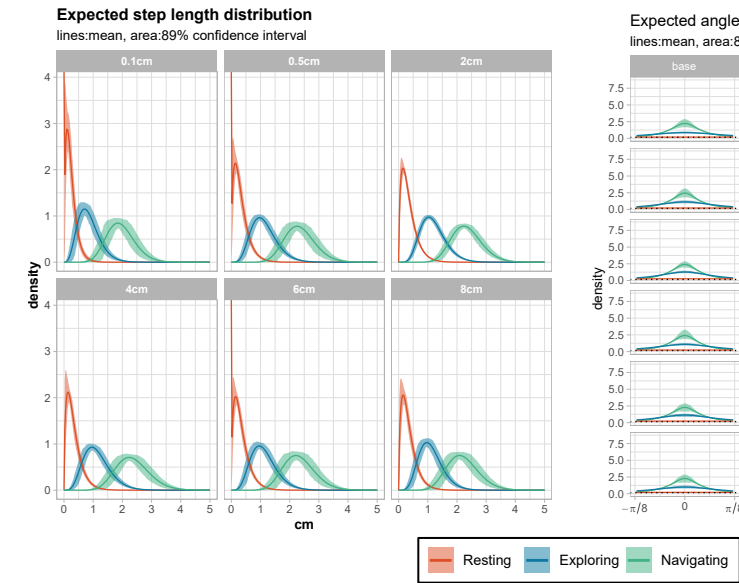

B

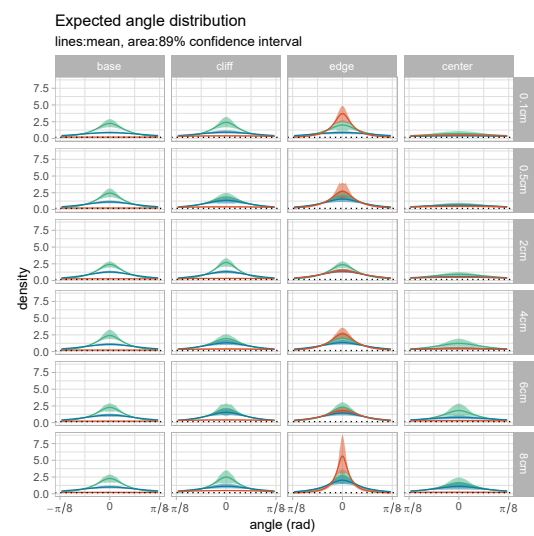

C

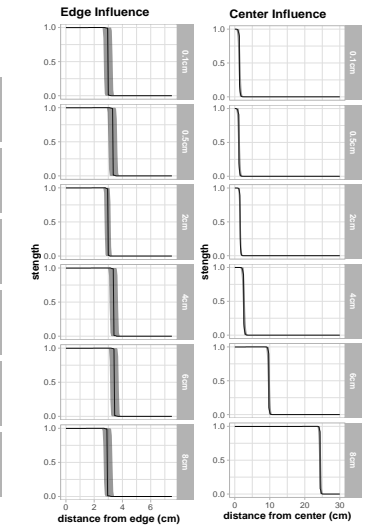

Contrast

D

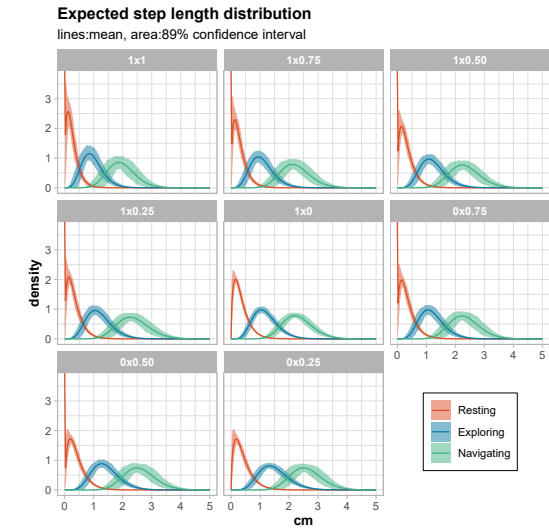

E

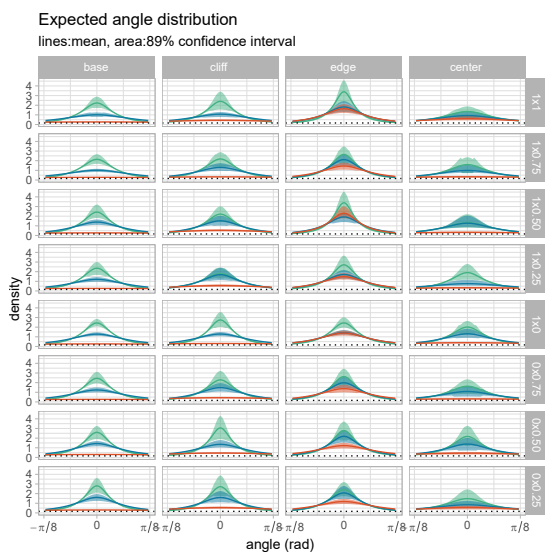

F

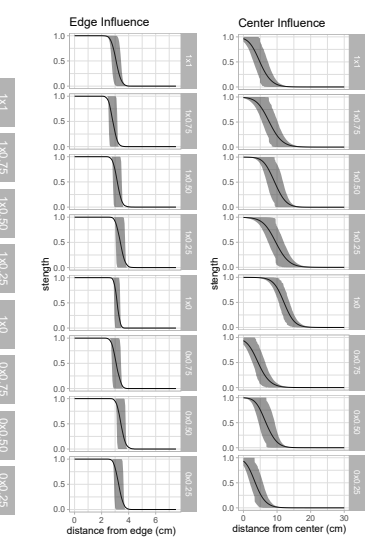

Time bin

G

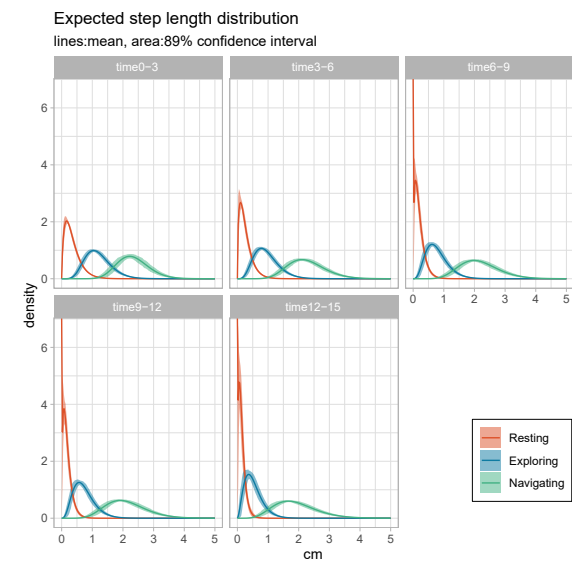

H

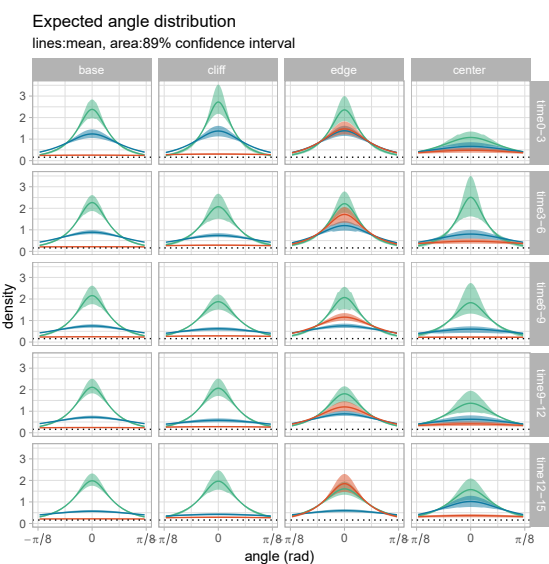

I

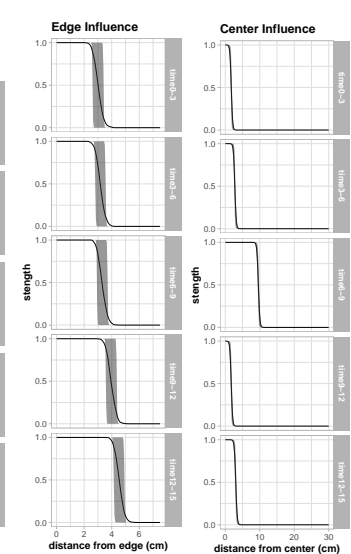

Supplement: S5 Fig — (A) Predicted distributions of step lengths for the three behavioral states (“Resting”, red; “Exploring”, blue; “Navigating”, green) across all pattern sizes. Solid lines represent the mean predicted distributions, while shaded regions indicate the 89% confidence intervals. Step lengths for the three states remain consistent across pattern sizes, with a stationary state (“Resting”) at approximately 0.1 cm, an intermediate exploratory state (“Exploring”) at ~1.1 cm, and a high-movement state (“Navigating”) at ~2.3 cm. (B) Predicted angular distributions for the three behavioral states under varying checkered pattern sizes. The angular range is restricted to [−π/8,π/8] to highlight differences in concentration across conditions. A dashed line indicates the uniform angle density (y=0.159) for reference. (C) Feature influence profiles for edge (left) and center (right) across pattern sizes. Edge influence remains stable across all conditions, with a consistent threshold at ~3 cm from the edge. Center influence, however, expands significantly with increasing pattern size. For smaller patterns (e.g., 2 cm), center influence diminishes ~4 cm from the center, whereas for larger patterns (6 cm and 8 cm), the influence extends outward, reflecting broader modulation of behavior. (D-E) Predicted distributions of step lengths (B) and angular differences (C) for each contrast condition. For step lengths, distributions of the three behavioral states (“Resting”, red; “Exploring”, blue; “Navigating”, green) are shown with lines representing the mean and shaded areas representing the 89% confidence interval. The angular distributions, restricted to the range [−π/8,π/8], illustrate the concentration of angular differences for basal conditions and near spatial features (cliff, edge, center). A dotted line indicates a uniform angular distribution (y=0.159). (F) Feature influence profiles for edge and center across contrast conditions. Edge influence (left) remains stable and robust, wit [file pone.0329367.s006.pdf]
