# Supplementary material for: Hidden Markov models reveal behavioral state dynamics in depth-related locomotion in mice
Source: PLoS One. 2025 Aug 26;20(8):e0329367. doi: 10.1371/journal.pone.0329367 (PMC12380309; doi:10.1371/journal.pone.0329367)
Supplement: S2 Fig — This figure presents the preliminary mixture model analysis conducted to determine the complexity required to represent angular and step length data from mouse trajectories. (A) Angular Data Fit: A histogram of observed angular differences (green) is overlaid with fitted two-component mixture models using wrapped Cauchy and von Mises distributions (purple lines). Both distributions capture the sharp central peak (minor directional adjustments) and the broad tails (larger directional shifts) present in the data. Single-component models were insufficient, necessitating at least two components to account for these distinct angular behaviors. (B) Angular Components: Estimated distributions of the two mixture components for angular data. The first component (red broader distribution) reflects exploratory movements, while the second (blue narrow peak near zero) represents minor directional adjustments. (C) Angular Mixing Ratios: Density plots (shaded areas) and points (individual-level estimates) illustrate the population-level mixture proportions (θ0) for the two angular models. The wrapped Cauchy model allocates approximately 20% to the broad component, whereas the von Mises model assigns ~40%, reflecting differences in how each model handles heavy tails. Although predictive performance differences were minor, the wrapped Cauchy model’s slight advantage informed our choice for HMM modeling. (D) Step Length Data Fit: A histogram of observed step lengths (green) is shown with fitted two- and three-component mixture models using Gamma and Lognormal distributions (purple lines). The step length data exhibit multiple peaks, and while two-component models improved upon single-component fits, a three-component Gamma model was necessary to fully capture the multimodal nature of movement intensities. (E) Step Length Components: The three-component Gamma model identifies distinct states of movement intensity: short step lengths (red, minimal movement), intermediate step lengths ( [file pone.0329367.s003.pdf]

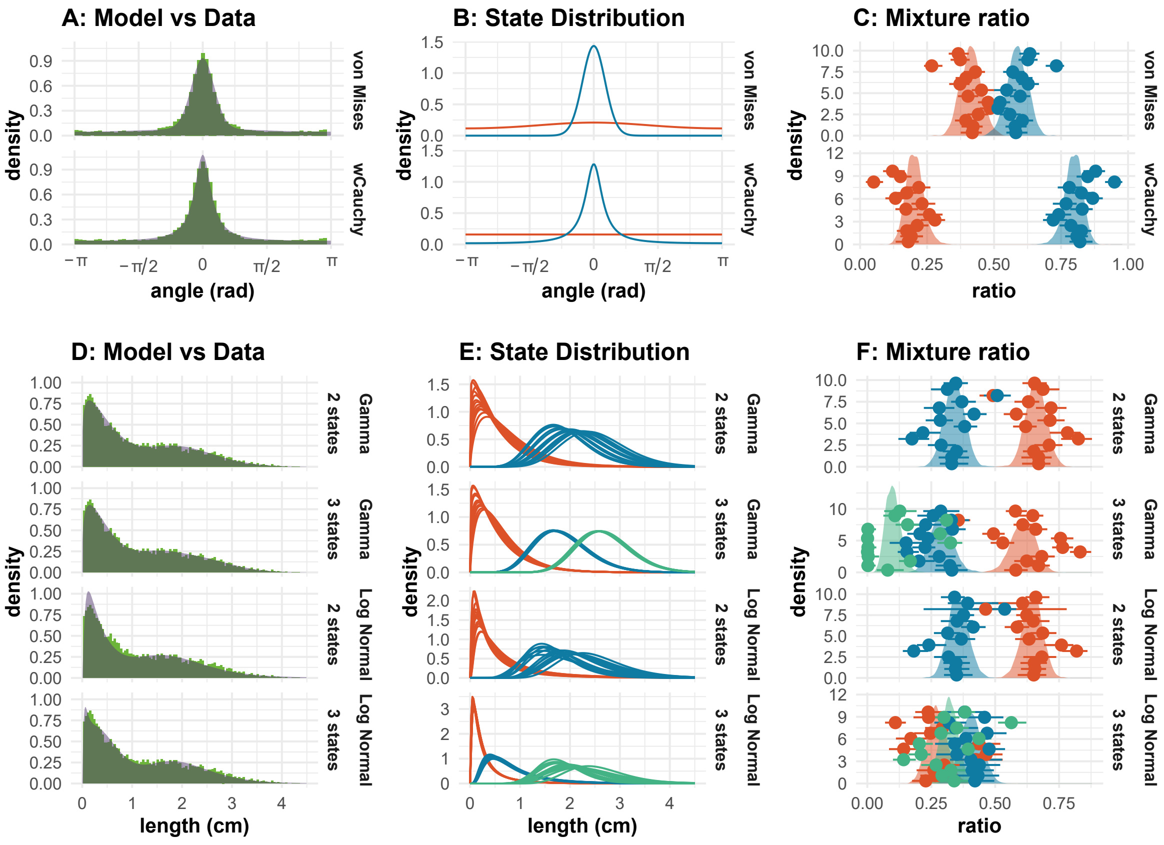

Supplemental Figure 2
