# Supplementary material for: Hidden Markov models reveal behavioral state dynamics in depth-related locomotion in mice
Source: PLoS One. 2025 Aug 26;20(8):e0329367. doi: 10.1371/journal.pone.0329367 (PMC12380309; doi:10.1371/journal.pone.0329367)
Supplement: S3 Fig — (top) Representative tracks of mice from three experimental groups: wild-type (WT), retinal degeneration model (RD), and wild-type in an open field setup (OF, no visual cliff). Tracks are color-coded to indicate continuous recordings, with each track representing the movement of an individual mouse over time. The tracks illustrate the spatial behavior of each group, with cliff locations indicated along the x-axis (cliff at x = 0x = 0x = 0). (bottom) Predicted behavioral states superimposed on the tracks. States are color-coded: “Resting” (red), “Exploring” (blue), and “Navigating” (green). The predicted states reveal the spatial distribution of behavioral patterns across the apparatus and highlight differences between experimental groups. WT tracks show a higher proportion of “Navigating” states near the cliff edge, indicating active exploration and interaction with the cliff. In contrast, RD mice display more “Resting” states throughout the apparatus, reflecting diminished visual input and exploratory behavior. OF mice exhibit a combination of “Exploring” and “Navigating” states, with reduced cliff-specific effects due to the absence of depth cues. (PDF) [file pone.0329367.s004.pdf]

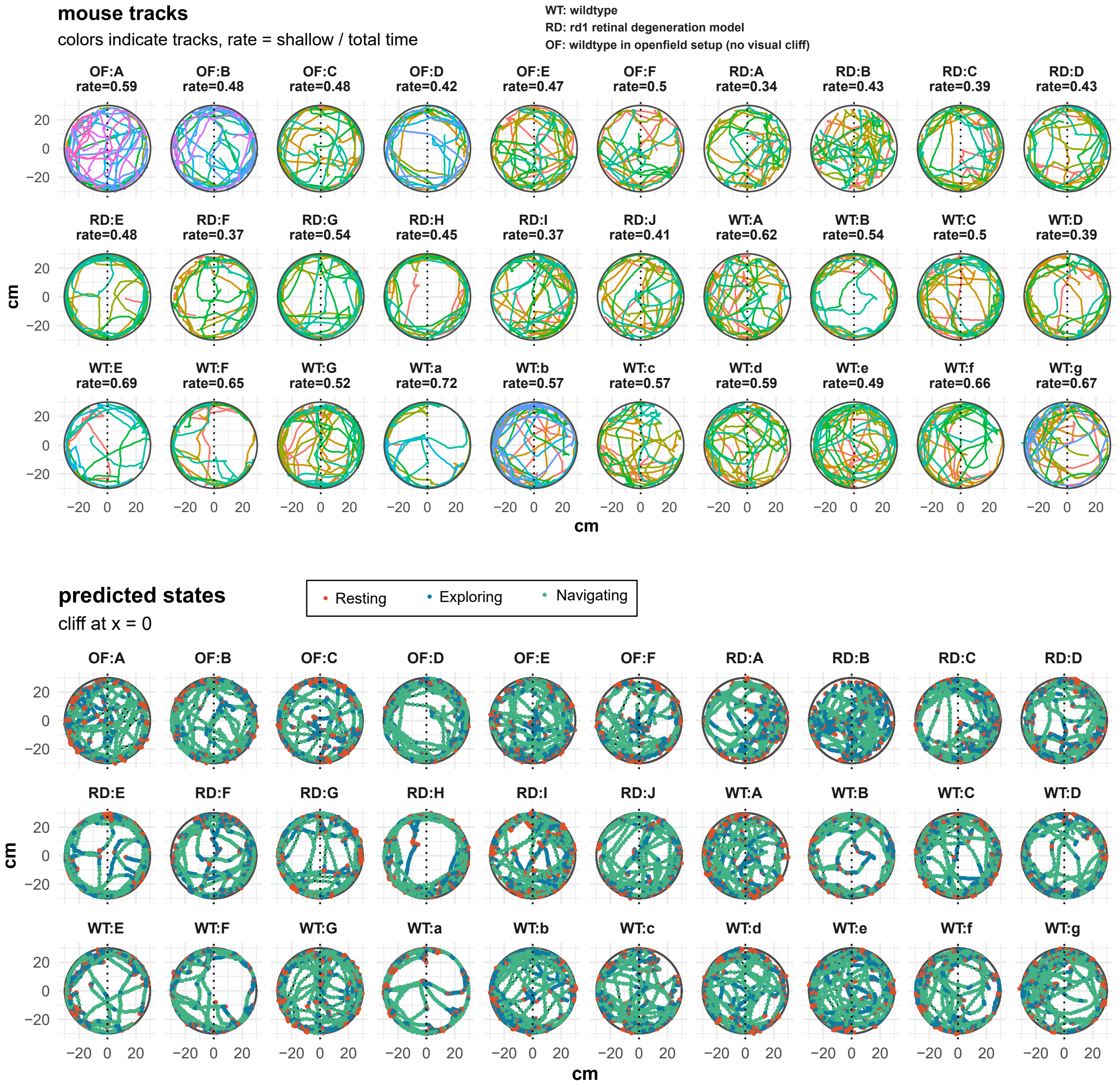

Supplemental Figure 3
