# Supplementary material for: Hidden Markov models reveal behavioral state dynamics in depth-related locomotion in mice
Source: PLoS One. 2025 Aug 26;20(8):e0329367. doi: 10.1371/journal.pone.0329367 (PMC12380309; doi:10.1371/journal.pone.0329367)
Supplement: Attachment — Submitted filename: Response to Reviewers.docx [file pone.0329367.s010.docx]

Response to Reviewers

# Responses to Reviewer #2

### Reviewer #2: The article is well-written and well-structured. The authors innovatively combined classic test models to avoid the limitations of using the models individually. Hence, the results of the authors' model can provide new inisght that help us understand the depth perception and related cognitive processes of mice. Good work! Before being considerated to be publihsed, the article still need some minor improvements concentrated on its writing and formality. Please see my comments below:

We thank the reviewer for their time, effort, and constructive feedback on our manuscript. We appreciate the positive comments regarding the overall structure, the innovative combination of classic test models, and the relevance of our results for understanding depth perception and related processes in mice. We have carefully considered the reviewer’s suggestions regarding minor improvements in writing and formality, and we have addressed each point below.

### Abstract: I am not sure about what the maximum keyword limit is. For clarity and to make it easier for readers to pinpoint the focus of the article, please remove some keywords that have overlapping meanings (in the scientific sense).

We have revised the keywords section by removing overlapping terms to enhance clarity and focus.

### Line 2-9: The first sentence of the abstract lacks clarity. You suggest that “Understanding how mice process and respond to visual depth cues is crucial for studying visual perception, but traditional behavioral analyses often miss subtle yet important aspects of this process”. What are the important aspects? How are those aspects related to the cues and processes you mentioned in the following sentences? Please clarify.

We appreciate the comment. We agree that the first sentence could be clearer regarding the important aspects of visual depth processing that traditional analyses might miss. We have revised the first sentence of the abstract to clarify that the important aspects include temporal dynamics, state transitions, and integration of multiple spatial cues—all of which are crucial for understanding how mice process depth information. We also now directly link these aspects to the methods and findings presented in the manuscript. This sentence was replaced with the following:

## “Understanding how mice process and respond to visual depth cues is crucial for studying visual perception, yet traditional behavioral analyses often miss key aspects of this process, such as the dynamic transitions between behavioral states, the influence of environmental context, and the integration of multiple spatial cues that shape depth-related behaviors. “

### Line 36-38: You suggest that “Simpler tests such as the optomotor response provide basic measures of visual acuity but fail to capture higher-order cognitive processes.” One should be cautious when using terms like cognitive or higher-order cognitive processes, which may imply intentionality and cross-species similarities.

We have revised the sentence to avoid implying intentionality or strong cross-species cognitive parallels, and to better align with a cautious, comparative perspective.

## Simpler tests such as the optomotor response provide basic measures of visual acuity but may not fully capture more complex behavioral adaptations and context-dependent responses.

### Line 130-132: You write “Our results demonstrate that this integrated approach provides deeper insights into visual processing than traditional methods, while offering a robust framework for quantitative behavioral analysis”. Using “while” in this context is not appropriate and causes confusion. Consider changing “while” to “and”.

We have replaced "while" with "and offer" to improve clarity in this sentence.

### Line 149: Consider adding photos of the apparatus.

A photograph of the apparatus is already included in Figure 1A. We have ensured that this figure clearly shows the apparatus as suggested.

### Start from line 134: Please note the inconsistencies of parameters (e.g., cm, mm, min). It is not appropriate to use abbreviations from the start without defining them. Instead, I suggest using the complete spelling of the parameters when they first appear, then adding abbreviations in brackets as a definition or explanation.

We revised all measurements to use millimeters (mm) for consistency, and defined all abbreviations at their first occurrence to ensure clarity throughout the manuscript.

### Line 159: Is “wildtype mice” an appropriate term? Please also include examples of species and their common and Latin names.

“Wildtype” is a commonly used term in the field, especially when contrasting it with the retinal degeneration model (RD). Nevertheless, we have revised the text to include the proper scientific names for both: C57BL/6J for the WT mice and rd1 for the RD mice.

## “Comparisons between wild-type C57BL/6J mice (Mus musculus), retinal degeneration models (rd1-2J, C57BL/6J background, Mus musculus), and control conditions confirm that these behavioral patterns specifically reflect visual processing rather than general exploratory behavior.”

# Responses to Reviewer #3

### The manuscript entitled "Hidden Markov Models Reveal Complex Depth Processing in Mouse Visual Behavior" presents a study that analyzes how mice adjust their locomotor behavior in response to visual cues of depth, using a circular open field setup and a probabilistic modeling approach. This is a thought-provoking and methodologically innovative article, with several relevant contributions, and I have genuinely enjoyed reading it.

### That said, I believe there are a number of aspects that could be reconsidered or further elaborated in order to deliver a more complete and balanced article for the readers of PLOS ONE. I outline these considerations below.

We appreciate the reviewer’s thorough and thoughtful comments, as well as their positive assessment of the methodological innovation and relevance of our study. We have carefully considered each of the reviewer’s suggestions and have made revisions to address the concerns and improve the clarity, rigor, and balance of the manuscript. Our point-by-point responses to the specific comments are provided below.

### Conceptual Framework and Title

### First, I would like to draw attention to the title of the article. Not because of its wording per se, but because it may point to a conceptual framing that could be refined. The phrase "…reveal complex depth processing" seems to imply a demonstration of internal perceptual or neural processes, yet the study does not directly measure perceptual mechanisms or neural responses. Rather, it investigates how behavior—specifically, the balance between exploratory and defensive responses—is modulated as the animal approaches a visually defined stimulus (the "cliff") in space. The title may therefore overstate the nature of the findings. A more accurate and transparent formulation would be one that reflects the behavioral and modeling focus of the work, such as highlighting state transitions, locomotor patterns, or probabilistic classification in depth-based tasks.

We appreciate this thoughtful comment. To better align the title with the study’s focus on behavioral state transitions and probabilistic modeling, we have revised the title from:

“Hidden Markov Models Reveal Complex Depth Processing in Mouse Visual Behavior”

to

“Hidden Markov Models Reveal Behavioral State Dynamics in Depth-Related Locomotion in Mice.”

We believe this new title more accurately reflects the study’s emphasis on state transitions and behavioral patterns rather than implying direct measurement of internal perceptual processes. Additionally, we have revised the short title from:

“HMM Analysis of Mouse Visual Depth Processing” to “HMM Analysis of Behavioral State Dynamics in Mice”.

We believe these changes align well with the reviewer’s suggestion to clarify the focus and avoid overstating the study’s claims.

### Along these lines, I believe the introduction could be strengthened by explicitly framing the study in terms of the functional interaction between exploratory and defensive tendencies. These behavioral systems are known to operate in tension, especially in ambiguous or risky environments. As the organism nears a potentially threatening stimulus, defensive responses tend to dominate; however, with repeated exposures, a degree of habituation can shift this balance back toward exploration. This dynamic is central to the interpretation of the results, and acknowledging it early on could help position the study within broader frameworks of behavior regulation, attention, and adaptation.

We agree with the reviewer’s suggestion to explicitly frame the study within the context of the interplay between exploratory and defensive behaviors. To address this, we have added a dedicated paragraph to the Introduction section that highlights how animals balance these behavioral systems, especially in ambiguous or risky situations such as approaching the visual cliff. This paragraph also discusses how repeated exposures can lead to habituation, shifting the balance back toward exploration. We believe this addition better positions our study within broader frameworks of behavioral regulation, attention, and adaptation, aligning with the reviewer’s insightful comment. We have added the following to the introduction

## “Importantly, animals constantly balance exploratory and defensive behaviors, especially in ambiguous or risky environments. When approaching a potentially threatening stimulus—such as the visually defined cliff—defensive responses tend to dominate. However, with repeated exposures, habituation may occur, shifting the balance back toward exploration. This dynamic interplay between behavioral systems is central to understanding how mice adapt their locomotor strategies in response to depth cues. By examining these state transitions, we can better interpret the observed behavioral signatures and situate them within broader frameworks of behavioral regulation, attention, and adaptation.”

### Experimental Design

### The circular open field design is a clear strength of the study. It elegantly avoids the corner biases that often confound results in square arenas and encourages more uniform spatial exploration. However, it would be helpful if the authors clarified whether this setup was controlled for potential confounds such as differences in lighting, floor reflections, or subtle asymmetries in visual patterns, which might influence movement patterns near the edges.

We acknowledge the reviewer’s point regarding potential confounds such as lighting, floor reflections, or subtle asymmetries in visual patterns in the circular open field setup. The square and circular setups were not conducted under strictly identical conditions, and therefore some potential for confounding exists. However, in our preliminary trials, we consistently observed corner-staying behavior in the square setup across various lighting conditions, while the circular setup consistently resulted in more uniform exploration regardless of the lighting conditions. We have added a note to the Methods section acknowledging this limitation and clarifying that the circular setup was found to reliably reduce corner-staying behavior across different experimental conditions.

## “Preliminary trials were conducted using both square and circular arena setups under varying lighting conditions to assess the potential influence of lighting, floor reflections, and subtle visual asymmetries on locomotor behavior. We consistently observed corner-staying behavior in the square arena regardless of lighting conditions, while the circular arena consistently promoted more uniform spatial exploration. Although the square and circular setups were not strictly matched for lighting or other visual features, the robust reduction in corner-staying behavior in the circular arena was consistent across these different conditions, suggesting that the arena shape was the primary driver of the observed behavioral differences.”

### Methodological Considerations

### Regarding the analysis method, the use of Hidden Markov Models (HMMs) is one of the most distinctive features of the study. Yet the rationale for choosing this specific approach could be articulated more explicitly. Since the identification of behavioral patterns depends to a great extent on the analytical framework employed, it is important for the reader to understand why this model was favored—particularly whether it was selected a priori, or after comparing it to other options. Were alternative unsupervised learning methods (e.g., k-means clustering, PCA-based trajectory analysis, or duration-aware segmentation models) considered and rejected? What specific advantage does the HMM provide in terms of interpretability or fit to the biological data? Addressing these questions would help readers appreciate the methodological decision and interpret the resulting "three-state" model with the appropriate caution.

We appreciate the reviewer’s suggestion to clarify the rationale behind our choice of Hidden Markov Models (HMMs). We selected the Bayesian HMM approach a priori, based on its flexibility and its capacity to incorporate probabilistic state transitions and continuous temporal dependencies—features that are well-suited to capturing the dynamic behavioral patterns observed in our experiments. Unlike alternative unsupervised learning methods such as k-means clustering or PCA-based trajectory analysis, HMMs allow us to model the probabilistic switching between behavioral states while accounting for the sequential nature of locomotor data.

Moreover, the Bayesian framework offers the added advantage of incorporating prior knowledge, which is particularly useful in behavioral experiments where certain environmental factors (such as distance to the edge or center) are known to influence movement. By integrating these covariates and setting reasonable priors, we can create a model that aligns with known behavioral tendencies while maintaining enough flexibility to capture unexpected patterns.

While we did not directly compare alternative unsupervised methods in this study, we acknowledge that different analytical frameworks could yield different state definitions. We have added a note in the Methods and Discussion sections to clarify that the three-state model reflects the chosen modeling approach and should be interpreted as model-dependent, not as a definitive classification of all possible behaviors.

We added this to the Methods section

## “We used a Bayesian Hidden Markov Model (HMM) to analyze locomotor behavior in the circular visual cliff arena. This approach was selected a priori because it captures the probabilistic switching between behavioral states and accounts for the sequential nature of locomotor data. The Bayesian framework allows us to incorporate reasonable prior knowledge about environmental factors (e.g., distance to the edge or center) that influence movement. By integrating these covariates and setting informative priors, we aimed to create a flexible model that reflects known behavioral tendencies while allowing for the detection of unexpected patterns. Although other unsupervised methods (e.g., k-means clustering, PCA-based trajectory analysis) exist, we chose the HMM approach because of its superior ability to handle temporal dependencies and interpretability in terms of state transitions. “

### Relatedly, it is worth noting that the categorization into three discrete states—resting, exploring, navigating—is a product of the model architecture itself. While these categories are intuitive and align well with observed behavior, it would be valuable to acknowledge that other methods might yield different numbers or types of states. This does not diminish the value of the results, but it does suggest that the findings should be interpreted as model-dependent, and potentially open to alternative decompositions of behavior. A brief discussion on this point would demonstrate awareness of the limitations of statistical segmentation and encourage future comparative validation.

We agree with the reviewer’s point that the three-state categorization is determined by the specific modeling approach employed and should not be interpreted as an absolute or definitive classification of mouse behavior. We have added a section to the Discussion explicitly acknowledging that alternative analytical frameworks might yield different behavioral states or segmentations. This clarification highlights that our results should be interpreted as model-dependent, and it underscores the importance of future work comparing different analytical approaches to better characterize behavioral dynamics in complex tasks.

The following paragraph was added to the discussion

## While our HMM approach addresses many limitations of traditional metrics, it also introduces specific challenges that affect the interpretation of visual processing behavior in mice. These limitations should be carefully considered when drawing biological conclusions.

## It is important to recognize that the three-state categorization (resting, exploring, navigating) produced by our HMM reflects the specific structure and assumptions of the model. Alternative analytical methods might yield different state definitions or numbers of states. Therefore, the states identified here should be viewed as one of several possible segmentations of locomotor behavior, contingent on the chosen analytical framework. Future work comparing different models may help refine these definitions and advance our understanding of behavioral state dynamics in complex tasks.

### Additional Methodological Questions

### Additionally, I would like to raise several specific questions that arose during my reading of the manuscript. While some of these may reflect gaps in my own understanding of the methodology, I believe they represent queries that could occur to readers of PLOS ONE, and addressing them explicitly in the text would enhance the manuscript's clarity and reproducibility.

### Methodological specifications:

### The rationale for tracking 12 body points when only the body center coordinates are used in the analysis could be clarified. Were the additional points considered for future analyses, or do they contribute to tracking accuracy?

In this study, the analysis was performed using only the body center coordinates. However, we found that using only the body center as a tracking label in DeepLabCut often resulted in unreliable tracking, likely due to limited visual information and the potential for occlusion. By labeling 12 body points—including head, tail, and limbs—DeepLabCut can more accurately estimate the body center coordinates by referencing the positions of all body parts. Although we did not use the coordinates of other body parts for analysis in this study, this comprehensive labeling approach improved the overall tracking quality and reliability of the body center data. Additionally, labeling 12 body points aligns with common practice in DeepLabCut, providing a standardized and robust framework for pose estimation.

### The 90% confidence threshold for DeepLabCut tracking appears somewhat arbitrary—was this threshold validated against manual scoring or compared to alternative cut-offs?

We acknowledge that the 90% confidence threshold for DeepLabCut tracking is somewhat arbitrary. However, preliminary analyses revealed that using cut-offs below 90% often included cases where the actual body part was not labeled correctly, which could compromise the accuracy of the analysis. To ensure high reliability in the extracted position data, we therefore chose to analyze only those data points with a confidence level of 90% or higher. Of all the tracked body parts, the body center consistently showed the highest confidence in labeling, reinforcing the decision to focus on this point for downstream analysis. While we did not systematically compare this threshold against manual scoring, this criterion provided a practical and effective balance between data quality and quantity in our study.

### While the 3000K lighting choice is explained by analogy to dawn/dusk conditions, it would be valuable to know whether this parameter was empirically validated or if alternative color temperatures were tested.

To determine the appropriate color temperature for our experiments, we conducted preliminary open field tests using three different color temperatures: 3000K, 6500K, and 8000K. We compared the total distance traveled by the mice under each condition and found no significant differences across the color temperatures, indicating that the mice maintained moderate activity levels regardless of the lighting. Based on these results, we concluded that color temperature did not have a substantial impact on locomotor activity in our setup. However, given that mice are crepuscular and naturally active during dawn/dusk periods, we selected 3000K as a naturalistic lighting condition to minimize potential stress and to better approximate their active phase. This choice aligns with previous literature on mouse activity patterns.

### Experimental design considerations:

### The protocol mentions that mice always start facing the shallow side—was counterbalancing of starting orientation considered to control for potential directional biases?

We did not consider counterbalancing the starting direction in this study. As the reviewer correctly points out, using the same starting direction for all mice may introduce a potential bias. However, our primary goal was to isolate the effects of visual cues on locomotor behavior under as consistent conditions as possible. Adding a parameter for starting orientation would have increased the complexity of the experimental design and potentially confounded the interpretation of the effects of visual depth cues. Therefore, we chose to have all mice start facing the shallow side to minimize variation in factors unrelated to vision.

### The procedure for "gently guiding" non-responsive mice could introduce variability—how frequently was this intervention required, and were these trials analyzed separately?

After reviewing all the video recordings, we found that only one RD mouse required gentle guidance to start the task. Given the very low frequency of this intervention and the sufficient amount of unaffected data, we did not analyze this trial separately. We believe that this single guided trial did not meaningfully impact the overall results or conclusions of the study.

### Results interpretation and presentation

### The authors acknowledge fixing certain spatial parameters (cliff influence at 6 cm) to prevent spurious effects in control groups. While this approach has merit, I wonder if it might limit the detection of genuine individual or group differences in spatial sensitivity to visual cues.

We agree that fixing the cliff influence parameter at 6 cm could potentially limit the detection of individual or group differences in spatial sensitivity. To address this, we ran several preliminary models using different values for the cliff influence parameter (ranging from 5 cm to 8 cm) and found that the results were virtually identical across this range. Based on this consistency, we chose to fix the parameter at 6 cm for simplicity and model stability. Ideally, we would have preferred to infer this parameter directly from the data; however, this approach introduced trade-offs with the center influence parameter, and we were unable to find a satisfactory prior distribution that allowed both parameters to be inferred simultaneously while ensuring model convergence.

### The discussion mentions that "dramatic cliff avoidance behaviors certainly occur" but are "relatively rare," which seems to contradict some of the stronger claims about depth processing complexity. Could the authors clarify how these infrequent but compelling behaviors relate to their overall model?

We acknowledge that dramatic cliff avoidance behaviors, while visually striking, were relatively rare events in our dataset. Our primary goal in this study was to capture more subtle and continuous changes in exploratory behavior that reflect how mice modulate their locomotion in response to depth cues. Rather than focusing solely on overt cliff avoidance, we analyzed behavior near the cliff edge in the context of other environmental factors (e.g., distance to the edge and center) to model these gradual shifts in behavior. This approach allowed us to demonstrate that mice respond to visual depth cues even when obvious avoidance behaviors are infrequent, thereby providing a more nuanced understanding of depth processing.

### The temporal analysis reveals interesting dynamics where the cliff effect diminishes and reappears over time. I wonder if this pattern might reflect habituation processes rather than sustained depth processing—could the authors comment on this interpretation?

### The behavioral state definitions appear to shift over time (e.g., state 2 at 12-15 min resembling early "Resting" more than "Exploring"). While the authors acknowledge this complexity, it might be helpful to discuss how this affects the biological interpretation of the states.

We agree that the temporal dynamics observed—where the cliff effect diminishes and reappears over time—are likely due to habituation rather than sustained depth processing. Indeed, in a separate study testing the response of mice to repeated exposures to the visual cliff, we found that after a couple of tests, mice rapidly habituate to the environment and exhibit similar state collapse behavior. In the current study, as shown in Supplementary Figure 5G, the three behavioral states were well separated in the early time periods (0-3 min and 3-6 min), but later (e.g., 12-15 min), the "Resting" and "Exploring" states converged and resembled each other, leading to a collapse toward two-state behavior. This dynamic highlights the importance of considering temporal context when interpreting the biological significance of the identified states. We have added this discussion point to the Discussion section to clarify that the identified states reflect both initial behavioral reactions to the cliff and subsequent habituation processes over time.

## The temporal dynamics analysis revealed perhaps our most surprising finding about visual processing: the evolution of behavioral responses over time suggests a sophisticated learning component to depth perception. Our analysis revealed that the temporal dynamics of behavioral states change over the course of the experiment, consistent with a process of habituation to the environment. Specifically, the initially distinct "Resting," "Exploring," and "Navigating" states began to converge after repeated exposure to the arena, with "Resting" and "Exploring" becoming less distinguishable in later time periods (e.g., 12-15 min; see Supplementary Figure 5G). This state collapse suggests that mice initially respond strongly to the cliff but gradually adapt their behavior as they become familiar with the environment. Such habituation processes are well documented in behavioral studies and highlight the need to interpret state transitions within the temporal context of the experiment. This also underscores the importance of considering both immediate and time-dependent factors when modeling behavioral dynamics in depth-related tasks.

### The integration of multiple spatial influences (cliff, center, edge) is intriguing, but the acknowledged difficulty in distinguishing between cliff and center effects raises questions about the specificity of the visual depth response.

We agree that distinguishing between cliff and center influences on behavior is challenging. We believe that cliff avoidance may be manifested in multiple ways, including subtle modulations of behavior near the cliff. Our open field tests (without the cliff) showed that when the cliff was absent, the influence of the center changed dramatically, suggesting that the presence of the cliff strongly affects how mice distribute their locomotion between the center and the edge. These observations support the idea that the cliff effect is not isolated but interacts with other spatial influences, making behavioral analysis inherently complex. We have added a note in the Discussion to acknowledge this complexity and clarify that our model captures these overlapping influences rather than attributing behaviors to a single factor.

We have added a note in the Discussion to acknowledge this complexity and clarify that our model captures these overlapping influences rather than attributing behaviors to a single factor.

## “We acknowledge that while our modeling captures both direct and indirect effects of the cliff, distinguishing between cliff-specific influences and center-based modulations is challenging. This overlap reflects the complex interplay of spatial factors that shape behavioral strategies in mice, and highlights the need for careful interpretation of model outputs when analyzing visually guided locomotion.”

### Some key findings rely on relatively small effect sizes (2-4% differences). While statistically robust, could the authors comment on the potential biological significance of these magnitudes?

We acknowledge that some of the observed effect sizes (e.g., 2–4% differences) may appear small. The visual cliff test is an experiment in which the data become meaningful by recognizing the cliff. However, our study suggests that some behaviors previously attributed to the cliff effect in traditional rectangular setups may actually result from mice’s inherent preference for corners. Given this insight, it is perhaps unsurprising that the behavioral changes attributable to the cliff effect alone are modest. While reviewing the video recordings, we did observe overt cliff avoidance behavior, but these were relatively rare, with mice often appearing “unaware” of the cliff. Nevertheless, we believe that detecting even a subtle but consistent cliff effect in this refined experimental design is a significant achievement.

Furthermore, as discussed in the manuscript, the cliff effect includes both direct and indirect components that manifest through influences on center and edge preferences. For example, the center influence was markedly different between WT (6.1 cm) and RD (23.6 cm) mice, and the edge influence similarly differed (WT: 3.2 cm; RD: 4.4 cm). When considered together, these spatial shifts represent meaningful behavioral adjustments that reflect the integration of multiple spatial cues. We have added a note to the Discussion to emphasize that even small effect sizes can be biologically meaningful in the context of subtle and complex behavioral adaptations.

## Comparative analysis of WT and RD mice provided additional insights into the role of vision in spatial behavior. As shown in Fig 4B, the effect size of the cliff perception between WT and RD mice was modest (2–4%). Although this may seem small, it captures behavioral changes that are highly dependent on visual input and demonstrates that vision does indeed impact depth perception. Importantly, even these small effect sizes were accompanied by clear differences in center and edge influences (Supplementary Figures 4G and 4H). For example, the center influence was substantially different between WT (6.1 cm) and RD (23.6 cm) mice, while the edge influence also differed between WT (3.2 cm) and RD (4.4 cm). When considered together, these differences suggest meaningful shifts in spatial behavior that extend beyond simple cliff avoidance. Rather than exhibiting random or undifferentiated movement patterns, RD mice displayed a fundamentally different pattern of spatial exploration, characterized by a broader center influence and reduced edge focus. These findings indicate that visual input not only enables depth perception but also shapes the underlying structure of exploratory behavior. This comparative analysis reveals that even modest effect sizes can reflect biologically significant differences in behavioral strategies, underscoring the complex and multifaceted role of vision in shaping spatial behavior.

### Given that many crucial results are presented in supplementary materials, readers might benefit from having some of these key findings highlighted more prominently in the main text.

Thank you for this suggestion. The Hidden Markov Model produces many parameters that are highly informative, and we initially presented these details in the supplementary materials for readers interested in the technical aspects of the analysis. However, we understand that highlighting some key findings more prominently in the main text would improve accessibility and readability. Therefore, we have revised the manuscript to incorporate clearer references to important parameters—such as the differences in center influence between WT and RD mice and the temporal collapse of behavioral states (habituation)—so that readers can more easily appreciate the significance of these findings without having to consult the supplementary materials.

### Summary

### In summary, this manuscript presents a compelling and well-executed study that combines a thoughtful behavioral design with a sophisticated modeling approach. The findings are original and relevant, particularly in their attempt to move beyond traditional binary behavioral metrics. I believe that with some refinement in how the framework is presented—both conceptually and methodologically—the article could reach a broader readership and have a greater impact. I hope the authors find these comments constructive in strengthening an already promising piece of work.

We would like to sincerely thank the reviewer for the thoughtful and constructive feedback. We have carefully addressed all the concerns raised, making substantial revisions to the manuscript to improve clarity, expand key points in the main text, and better integrate important methodological and conceptual details. We hope that these revisions meet the reviewer’s expectations and enhance the overall quality and accessibility of the study.
